# There is a paucity of economic evaluations of prediction methods of caries and periodontitis—A systematic review

**DOI:** 10.1002/cre2.405

**Published:** 2021-02-16

**Authors:** Helena Fransson, Thomas Davidson, Madeleine Rohlin, The Foresight Research Consortium, Helena Christell

**Affiliations:** ^1^ Faculty of Odontology, Department of Endodontics Malmö University Malmö Sweden; ^2^ Department of Endodontology, Institute of Odontology The Sahlgrenska Academy, University of Gothenburg Gothenburg Sweden; ^3^ Department of Medical and Health Sciences, Centre for Medical Technology Assessment Linköping University Linköping Sweden; ^4^ Faculty of Odontology, Department of Oral Biology Malmö University Malmö Sweden; ^5^ For a listing of the consortium partners visit: https://mau.se/en/research/research-programmes/foresight/; ^6^ Department of Radiology Helsingborg hospital Helsingborg Sweden

**Keywords:** cost effectiveness, economics, risk assessment, systematic review

## Abstract

**Objectives:**

Direct cost for methods of prediction also named risk assessment in dentistry may be negligible compared with the cost of extensive constructions. On the other hand, as risk assessment is performed daily and for several patients in general dental practice, the costs may be considerable. The objective was to summarize evidence in studies of economic evaluation of prognostic prediction multivariable models and methods of caries and periodontitis and to identify knowledge gaps (PROSPERO registration number: CRD42020149763).

**Material and methods:**

Four electronic databases (PubMed, Web of Science, The Cochrane Library, NHS Economic Evaluation Database) and reference lists of included studies were searched. Titles and abstracts were screened by two reviewers in parallel. Full‐text studies reporting resources used, costs and cost‐effectiveness of prediction models and methods were selected and critically appraised using a protocol based on items from the CHEERS checklist for economic evaluations and the CHARMS checklist for evaluation of prediction studies.

**Results:**

From 38 selected studies, six studies on prediction fulfilled the eligibility criteria, four on caries and two on periodontitis. As the economic evaluations differed in method and perspective among the studies, the results could not be generalized. Our systematic review revealed methodological shortcomings regarding the description of predictive models and methods, and particularly of the economic evaluation.

**Conclusions:**

The systematic review highlighted a paucity of economic evaluations regarding methods or multivariable models for prediction of caries and periodontitis. Our results indicate that what we currently practice using models and methods to predict caries and periodontitis lacks evidence regarding cost‐effectiveness.

## 1 INTRODUCTION

Caries and periodontitis are among the most prevalent oral diseases. The distribution and severity vary between individuals within the same region or country (Petersen et al., 2008) resulting in a skewed distribution of disease. The challenge today is to identify individuals not at risk and those with increased risk for future disease development. Identification of individuals not at risk, will enable reduced redundant recalls and examinations in the dental practice. For those with an increased risk tailor‐made recalls and interventions will be enabled.

The identification of individuals with an increased risk of developing caries lesions using prognostic assessments, also called risk assessment, is either based on a method with one single variable, for example, previous caries experience, number of microbiota, salivary buffering capacity or salivary flow rate (Mejàre et al., 2014; Senneby et al., 2015), or on multivariable prediction models such as the Cariogram (Bratthall & Hänsel‐Petersson, 2005) and Caries Management by Risk Assessment (CAMBRA; Featherstone et al., 2003). To predict the progression of periodontal disease, the presence or absence of bleeding on probing (Lang et al., 1990) or probing pocket depth (Haffajee et al., 1991) has been applied as single methods. For the risk assessment models of periodontal disease, age, smoking and diabetes status are most commonly included (Du et al., 2018).

The direct cost for a method or model used for risk assessment in dentistry may be negligible compared to the cost of extensive constructions. On the other hand, as risk assessment and decisions about recalls and intervals between examinations as well as interventions are performed for several patients daily in general dental practice, a comprehensive cost has to be taken into account, which from a societal perspective, means to allow for effective resource allocation. However, the societal cost of a method may not equal the cost that the clinicians face. Hence, health economic evaluation that is a set of formal methods is applied to analyze resource use and outcomes of interventions such as diagnostics, prediction, prevention and treatment. As public resources are limited, they should ideally only be used in programmes that are cost‐effective. A recent scoping review indicated that the number of economic evaluations in dentistry is increasing (Eow et al., 2019) but the need is still great and thus more work in this field is necessary. The results of a systematic review of economic evaluation demonstrated that evidence is limited for diagnostic methods used in dentistry (Christell et al., 2014). Similar results may be assumed to apply for methods used for prognostic prediction.

The purpose of a systematic review (SR) is to provide evidence for an intervention based on the summary of current literature and to identify knowledge gaps to guide future research within a knowledge field. In SRs that include economic evaluations, information is compiled not only on whether interventions work but also on the economic aspects of the actual interventions. The objective of this SR was to further the understanding in this field by collecting data of studies of economic evaluations of prognostic multivariable models and methods for prediction of caries and periodontitis by using a SR approach.

## 2 METHODS

Based on current method guidelines (Moher et al., 2010) we reviewed the principal findings of economic evaluations of multivariable models and methods for prognostic prediction of increased risk of caries as well as of periodontitis. To ensure a systematic approach, the SR was structured in the following steps: (a) formulating the review questions, (b) formulating eligibility criteria, (c) identification and selection of studies, (d) collection of data and appraisal of included studies and (e) synthesis and interpretation. PRISMA statement (Shemilt et al., 2019) was implemented, and the SR was registered in PROSPERO (International prospective register of systematic reviews; PROSPERO, 2020) as CRD42020149763.

### 2.1 Review questions

The SR of the literature on economic evaluations of multivariable models and methods for prognostic prediction of the increased risk of caries and periodontitis aimed to address the following questions:

- Which multivariable models and methods for prognostic prediction have been analyzed?
- What are the estimated resources used, costs and/or cost‐effectiveness of the multivariable models and methods?
- What are the potential strengths and weaknesses of the methodologies of the included studies?
- To what extent may the evaluations help decision‐makers to direct resources efficiently?

The following elements were defined prior as:

- Economic evaluation: either full economic evaluation according to the definition by Husereau et al. (2013) as a comparative analysis of alternative courses of action in terms of costs (resource use) and consequences (outcomes, effects) or a partial economic evaluation defined as cost analyses and cost‐description studies (Moher et al., 2010).
- Multivariable model: a model based on a mathematical equation that relates to at least two variables (Moons et al., 2014).
- Prediction model: a tool that combines multiple predictors by assigning relative weights to each predictor to obtain a risk or probability. They are developed to estimate the probability or risk that a specific disease or condition is present (diagnostic models) or that a specific event will occur in the future (prognostic model; Moons et al., 2014).
- Prognostic prediction: an estimation of the probability that an individual will experience a specific event or outcome within a certain time period (Collins et al., 2015).
- Risk: this is defined as the probability of an unwanted event that may or may not occur. Risk of dental caries is a probable development from either (a) a sound tooth surface to a lesion in enamel or dentin, that is, from health to disease or (b) an existing lesion to a more extensive lesion, that is, from disease to more severe disease (Senneby et al., 2015). The risk of periodontitis is a probable development from either (a) sound gum to bleeding on probing, increased pocket depth and loss of alveolar bone height or (b) increased pocket depth and loss of alveolar bone height to bleeding on probing, further increase of pocket depth and loss of alveolar bone height.

### 2.2 Eligibility criteria

Eligibility criteria were determined a priori according to the review questions and based on PIRO (Population, Index test; Reference standard, Outcomes):

*Inclusion criteria*: included studies had to meet a minimum set of criteria concerning

- Population: humans of all age groups.
- Index test: clinically applicable multivariable model or method for prognostic prediction of caries and caries development or of periodontal disease.
- Reference standard:
  - Caries progression also named caries increment/incidence/experience presented at an individual basis.
  - Progression of periodontal disease and/or tooth loss at an individual basis.
- Outcomes of index test (predictive performance):
  - Predictive accuracy efficacy expressed as sensitivity, specificity, positive and negative predictive values, positive and negative likelihood ratios, area under the receiver operating curve (AUC) or data required to populate 2 × 2 tables cross‐classifying index test and reference standard.
  - Consequences of the prediction of progression of caries or periodontal disease such as recalls for examination.
- Outcomes of economic evaluation: resources use, costs or cost‐effectiveness data.
- Language: studies with English abstracts and written in English or German would be considered for inclusion in a pragmatic way, based on the availability of adequate translation skills within the authors' institutions.

*Exclusion criteria*: Studies were excluded if they contained no data of either multivariable model or method for prognostic prediction of caries and caries development or of periodontal disease together with an economic evaluation. Narrative reviews, editorials, case reports and individual (clinical) studies were excluded.

### 2.3 Identification and selection of studies

The electronic databases searched were: MEDLINE via PubMed, the Web of Science, the Cochrane Library and the NHS Economic Evaluation Database (NHS EED) until it stopped at the end of 2014. MESH‐terms as well as free‐text terms were combined (Table S1). Search strings were developed together with librarians and adapted for use across databases, combining the MeSH using the “AND” operator with index terms (Table S1). The publication years ranged from database inception to September 2, 2020. In addition, the reference lists of included publications were screened for publications not captured by the electronic searches. We also searched the PROSPERO database (terms: economic evaluation/cost‐effectiveness of prognostic prediction/predictive methods/risk‐assessment of caries/periodontitis) on August 26, 2020 to allow identification of any forthcoming studies.

The selection of studies was completed in two phases. In phase‐one, the retrieved records were assessed according to title and/or abstract by two reviewers in parallel and selected according to the review questions and eligibility criteria. Records selected by at least one reviewer were retrieved in full‐text for further assessment. In phase‐two, two reviewers in parallel applied the same eligibility criteria to the full‐text studies. Any disagreement was resolved by consensus among the reviewers.

### 2.4 Collection of data and appraisal of included studies

Four reviewers, one of them being a health economist, were involved in the data collection. A table for data collection regarding both the prognostic prediction method/model and the economic evaluation was developed a priori and piloted by the four reviewers in parallel using five studies. Discussion was held among the reviewers and discrepancies were resolved until consensus was reached. The data on the prediction method/model of each study considered to be important was the description and the predictive performance of the methods/model. For the economic evaluation data as proposed by Shemilt et al. (2019) was collected when available on (a) the analytic framework and type of economic evaluation, (b) the analytic perspective (whose costs and benefits a decision maker views as important) and time horizon, (c) main cost items includes in the analysis and (d) the setting (i.e., country, health care system), currency and price year. Data from included studies were tabulated by one reviewer and checked by the other reviewers, who read the studies in parallel to verify the accuracy of collected data and to ensure that relevant data was included. Any disagreement was resolved by consensus among the four reviewers. When predictive performance was not presented in the included study, data required populating tables cross‐classifying index test and reference standard was searched in studies referenced to, enabling the calculation of predictive performance.

For the critical appraisal, a protocol was designed and applied in included studies. Items from CHARMS checklist for reviews of prediction modeling studies (Moons et al., 2014) and checklist for reporting economic evaluations (CHEERS; Husereau et al., 2013) were included. The protocol contains five domains with signaling questions: sample, prediction model or method, outcome to be predicted, economic evaluation, and analysis and interpretation of results (Table 1). Each signaling question is formulated to be answered by one of “Yes,” “No,” “Unclear” or “Not applicable.” First, the four reviewers assessed included studies in parallel and then compared the answers of each signaling question. Discrepancies regarding answers for the signaling questions were discussed until consensus was reached.

**TABLE 1:** Tool for critical appraisal of studies of economic evaluation of prognostic multivariable methods and models for prediction of caries and periodontitis

First author						
Journal/year/volume/pages	Evaluated by	Date	Yes	No	Unclear	NA
*Domain 1*: *Sample*						
Was target population to whom prediction method/model applies described?						
Was setting of recruitment described (e.g., primary care, secondary care, general population)?						
Were number, age and sex of included individuals presented?						
Was disease prevalence of included groups presented?						
Was study dates presented?						
*Domain 2*: *Method/model for prediction of caries and/or periodontitis (index test)*						
Was method for prediction described to permit replication?						
Was model with types and handling of predictors described to permit replication?						
Were examination method(s) and measurements presented?						
Was number of examiners and their experience described?						
If implemented was thresholds clearly described?						
Was timing of measurement of predictor(s) described?						
*Domain 3*: *Outcome to be predicted (reference standard) and method/model performance*						
Was type of outcome(s) to be predicted defined?						
Were outcome thresholds and criteria for thresholds clearly described?						
Were examination method(s) and measurements presented?						
Was outcome assessed without knowledge of candidate predictors (blinded assessment)?						
Was performance of model or method presented adequately?						
*Domain 4*: *Economic evaluation*						
Was the study design appropriate?						
If simulation was used was the model transparent?						
Was the perspective of the evaluation presented?						
Were all relevant costs identified, presented and valued?						
Was time horizon long enough to reflect all relevant costs and effects?						
Were outcome measures captured and quantified for all important effects?						
Was discount rate(s) stated?						
Was uncertainty considered?						
*Domain 5*: *Analysis and interpretation of results*						
Was the result presented as costs related to effects (ratio)?						
Was sensitivity analysis with relevant variables performed?						
Is the result robust in regard to results of sensitivity analysis?						
Is the result transferable to other settings?						
Is the analysis helpful for a decision‐maker?						
*Comments*						
Not applicable						

### 2.5 Analysis and interpretation

A narrative approach was used to present the analysis of collected data of the estimated resources used, costs and/or cost‐effectiveness. Based on the collected data and answers to the signaling questions of the five domains presented in Table 1, an overall judgment of “Strengths” and “Weaknesses” of included studies was made by the four reviewers jointly. The generalizability and transferability of each economic evaluation was interpreted when answering the review question “To what extent can the evaluations help decision‐makers to direct resources efficiently?”

## 3 RESULTS

### 3.1 Identification and selection of studies

**FIGURE 1:**
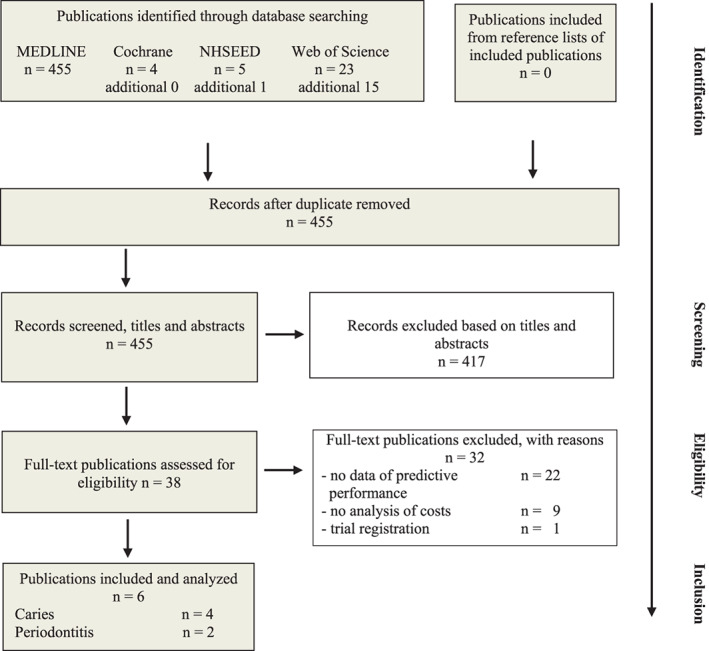
Flow diagram according to PRISMA Statement (Shemilt et al., 2019) presenting results of searches performed on September 4, 2019 and up‐dated September 2, 2020 and study selection

As presented in Figure 1, 455 records were identified after removal of duplicates, 38 full‐text publications were read, and 32 full‐text publications were excluded because they contained no data on predictive performance or of an economic evaluation (Table S2). Six studies, published between 1994 and 2014 and listed in Table 2, were included.

**TABLE 2:** Characteristics of included studies on economic evaluation of prognostic prediction methods and multivariable models of caries or periodontitis

Reference	Sample	Prognostic prediction method and model		Economic evaluation			Results by authors
	Number (*n*) Age (year) Setting	Method/model Follow‐up time	Predictive performance	Study design	Type of evaluation: stated by authors as defined by Husereau et al. (2013)	Costs presented as	
*Prognostic prediction method for caries*							
Jokela and Pienihäkkinen (2003)	Group I Risk‐based prevention *n* = 299 Group II Routine prevention *n* = 226 2 years One group in each of two municipal health centers (1989–1993)	Group I: risk‐based prevention based on dental assistants' screening of *mutans streptococci* (MS) in proximal plaque (Dentocult SM® Strip Mutans) and incipient or other carious lesions low risk: MS‐free and caries‐free intermediate risk: MS‐positive with no caries high risk: signs of caries Group II: routine prevention based on dentists' decisions. Follow‐up time 3 years	Performance^a^ to predict presence of cavitated lesions or fillings: *Sensitivity*: low‐risk versus intermediate + high‐risk 0.72 low‐risk + intermediate versus high‐risk 0.32 *Specificity*: low‐risk versus intermediate + high‐risk 0.77 low‐risk + intermediate versus high‐risk 0.98	Empirical study	Cost analysis Cost analysis	Mean running costs in Euros (€)	Cost per child for 3‐year time for examination, prevention and treatment: Group I 54€ Group II 69€ (Student's *t*‐test *P* = 0.004) Risk‐based prevention can be effective in reducing both costs and dental caries in preschool children when screening and preventive measures are delegated to dental assistants
Zavras et al. (2000)	*n* = 1180 1 or 2 years Community‐based private pediatric dental practice (1988–1995)	Group I: microbiological screening of salivary MS recorded as colony‐forming units (CFU) counts Group II: no screening Follow‐up time 1 or 2 years NR	Predictive performance based on CFU levels (low, moderate, high, too numerous to count) to predict caries: *Sensitivity* at 1 year 0.37–0.66 at 2 years 0.34–0.72 *Specificity* at 1 year 0.96–0.88 at 2 years 0.96–0.81	Model study	Cost analysis Cost analysis	Cost in US$ Costs based on fees for New England dental insurers	Cost for predictive method 20$ Total cost per child Group I 367.90$ Group II 396.70$ Cumulative dental treatment cost for a child aged 4 years lower if the child was screened Caries prevalence 15% (range 5%–51%) Cost savings increase significantly when caries prevalence increases
*Multivariable model for prediction of caries*							
Holst and Braune (1994)	*n* = 102 2–4 years One test clinic in public dental health (1987–1991) versus. all public dental health clinics in one county (1991)	Risk assessment in test clinic based on factors given different weights: health status, medication, eating and drinking habits, oral hygiene, use of fluorides, parents knowledge of caries, parents interest in given information, visible caries Follow‐up times: from 2 to 4 years from 3 to 4 years Risk‐assessment by dental assistants and follow‐up examination by dentist	Predictive performance of risk assessment for manifest caries lesion: *Sensitivity* at 2–4 years 0.42 at 3–4 years 0.58 *Specificity* at 2–4 years 1.0 at 3–4 years 0.99	Empirical study	NR Cost‐effectiveness analysis	Mean time (min) spent per child up to 4 years at test clinic compared with mean time per child at clinics of the whole county based on county epidemiology	Mean value for time spent (min) at: test clinic 27 for dentist and 71 for dental assistant county clinics 60 for dentists and 90 for dental assistants Time spent was 50 min less in test clinic Caries prevalence 19% at test clinic and 23% at county clinics Test model for caries prevention is cost‐effective
Holst et al. (1997)^b^	*n* = 99 2–4 years *n* = 102 3–4 years One test clinic in public dental health (1990–1994) versus all public dental health clinics in one county (1994)	Risk assessment based on any single factor: illness for 1 week more than four times a year, saliva inhibiting drug, six daily intakes of food/drinks, anything else but water at night, oral hygiene less than once a day, no fluorides, visible plaque, visible caries Follow‐up times: from 2 to 4 years from 3 to 4 years Risk assessment by a dental assistant and follow‐up examination by dentist	Predictive accuracy of risk assessment for manifest caries lesions: *Sensitivity* at age 2–4 years 1.0 at age 3–4 years 0.86 *Specificity* at age 2–4 years 0.70 at age 3–4 years 0.66	Empirical study	NR Cost‐effectiveness analysis	Mean time minutes (min) spent per child up to 4 years at test clinic compared with mean time per child at county clinics based on county epidemiology	Mean value ‐time spent (min) at: test clinic 14 for dentist and 152 for dental assistant county clinics 42 for dentist and 102 for dental assistant Time spent for dentist was 28 min less in test clinic Caries prevalence 7% at test clinic and 24% at county clinics Test model for caries prevention is cost‐effective
*Multivariable model for prediction of periodontitis*							
Higashi et al. (2002)	Hypothetical cohort with patients 35 years with mild periodontitis representing eight sub cohorts based on: treatment/no treatment smoker/non‐smoker Interleukin‐1 (IL‐1) genotype positive/negative Setting: periodontist specialist clinic	IL‐1 test (positive or negative) Follow‐up time: 30 years Periodontist	Predictive accuracy used for modeling assumption to identify patients with high risk for progression to severe periodontal disease: PPV 0.97 (range 0.94–1) NPV 0.97 (range 0.94–1)	Model study based on decision‐analysis and Markov modeling	Cost‐effectiveness analysis Cost‐utility analysis	Cost in US$ per Quality‐Adjusted Life‐Year (QALY)	Calculations of cost for genetic test 218$ Use of test compared with no‐test resulted in additional cost of 147,114$ per 1000 patients over a 30‐year time frame Reduction of number of cases with severe periodontitis 6.1 (absolute decrease 0.61%) QALYs increased by 4.5 using test Genetic test compared to no‐test ICER 32,633$ per QALY gained
Martin et al. (2014)	Group I patients receiving periodontal treatment *n* = 776 mean age 46 years (range 19–84) (1971–2003) Group II patients receiving routine dental care *n* = 523 males mean age 47.3 years (range 28–71) (1968–1988) Setting: private dental clinics	Chronic periodontitis (CP) risk score based on following factors: patient age, periodontal disease severity (deepest pocket, bleeding on probing, greatest radiographic bone loss), smoking history, diabetic status, periodontal treatment history, furcation involvements, vertical bone lesions, subgingival calculus or restorations Risk on a scale of 1 (very low risk) to 5 (very high risk) for alveolar bone loss and tooth loss Follow‐up: 13 years NR	Prediction of tooth loss^c^: Score 2 versus 3, 4, 5: *Sensitivity* 0.92 *Specificity* 0.33 PPV 0.59 NPV 0.80 LR+ 1.4 LR− 0.25 Score 2, 3 versus 4, 5: *Sensitivity* 0.60 *Specificity* 0.78 PPV 0.71 NPV 0.64 LR+ 2.7 LR− 0.51 Score 2, 3, 4 versus 5: *Sensitivity* 0.32 *Specificity* 0.93 PPV 0.83 NPV 0.56 LR+ 4.8 LR− 0.72	Model study	Cost–benefit analysis Cost‐effectiveness	Cost in US$ of periodontal treatment to preserve one tooth related to risk score and severity of CP	For high or moderate risk combined with any severity of CP, cost of periodontal treatment divided by number of teeth preserved ranged from 1405 to 4895$ Periodontal treatment is justified on basis of tooth preservation when risk is moderate or high regardless of CP severity For low risk with mild CP, cost of periodontal treatment is higher than fixed replacement

Abbreviations: ICER, incremental cost‐effectiveness ratio; LR, likelihood ratio; NPV, negative predictive value; NR, not reported; PPV, positive predictive value.

^a^ In detail in Pienihäkkinen and Jokela (2002).

^b^ Similar model as in study above. Different factors and extended sample.

^c^ According to our calculations based on Fig. 4 in Page et al. (2003).

### 3.2 Which multivariable models and methods for prognostic prediction have been analyzed?

Table 2 presents the methods and multivariable models for prognostic prediction of caries or periodontitis of the included studies. Four studies presented the prediction of caries in children aged 1–3 years with a follow‐up of 1–3 years, two studies on methods (Jokela & Pienihäkkinen, 2003; Zavras et al., 2000) and two studies on multivariable models (Holst et al., 1997; Holst & Braune, 1994). The methods were based either on the amount of salivary *mutans streptococci* as the predictor (Zavras et al., 2000) or the presence of caries lesions together with the presence of *mutans streptococci* (Jokela & Pienihäkkinen, 2003 *)*. The studies of multivariable models for the prediction of caries (Holst et al., 1997; Holst & Braune, 1994) were based on several factors (*n* = 7 or 8) given different weights. Two studies presented the prediction of periodontitis or tooth loss due to periodontitis in middle‐aged persons with a follow‐up of 13 and 30 years, respectively. In one study (Higashi et al., 2002), the prediction was based on a model with a biomarker (interleukin‐1), whilst the other study presented a model based on risk scores containing nine factors (Martin et al., 2014). Predictive performance was presented as sensitivity, specificity or predictive positive or negative values. For one study (Martin et al., 2014), we calculated the predictive performance based on a figure presented in a referred study (Page et al., 2003).

### 3.3 What are the estimated resources used, costs and/or cost‐effectiveness of the multivariable models and methods?

The estimated costs and/or cost‐effectiveness of each study are presented in Table 2. Two studies (Jokela & Pienihäkkinen, 2003; Zavras et al., 2000) were cost analyses and hence only presented the costs, while the other four (Higashi et al., 2002; Holst et al., 1997; Holst & Braune, 1994; Martin et al., 2014) presented both costs (resources used) and outcomes. In two studies (Holst et al., 1997; Holst & Braune, 1994), the costs were constituted by the resources used at the clinics, presented as mean time spent per child, and the outcomes were measured as the difference in caries prevalence at follow‐up between the test clinic and the comparator clinic. One study used QALYs as outcome (Higashi et al., 2002), where QALY weight decrements of 0.02 and 0.07 were used for mild and severe periodontitis, respectively. In one study number of teeth preserved was used as an outcome measure (Martin et al., 2014).

Four studies (Holst et al., 1997; Holst & Braune, 1994; Jokela & Pienihäkkinen, 2003; Zavras et al., 2000) showed cost savings due to the use of methods or multivariable models for the prediction of caries; hence, they were assumed to be cost‐effective. The other studies (Higashi et al., 2002; Martin et al., 2014) showed increased costs but improved outcomes. Whether they were considered cost‐effective depends on the willingness‐to‐pay of the decision‐maker.

### 3.4 What are the potential strengths and weaknesses of the methodologies of the included studies?

Table 3 presents the strengths and weaknesses of each included study. Samples concerning target population, setting and time of recruitment, number, age and sex of included participants, disease prevalence at baseline were reported adequately in most studies. Several shortcomings were found regarding the predictive method/model and in particular for the economic evaluation. Overall, the prognostic prediction method/model was described, but not to permit replication. There was an incomplete description of the examination methods and measurements and of the outcomes to be predicted. Also, the economic evaluations were often not completely performed or reported, for example, regarding the perspective of the analysis and sensitivity analyses, but the results were most often clearly reported regarding the main outcome.

**TABLE 3:** Critical appraisal of included studies on economic evaluation of prognostic prediction methods and multivariable models of caries or periodontitis

	Strengths			Weaknesses		
Reference	Prediction method/model and Outcome to be predicted	Economic evaluation	Analysis and interpretation of results	Prediction method/model and Outcome to be predicted	Economic evaluation	Analysis and interpretation of results
Jokela and Pienihäkkinen (2003)	*Prediction method*: definition, methods to assess, thresholds of outcomes presented *Outcome to be predicted*: type of outcome and predictive performance presented	Cost‐analysis with cost for labor and material Perspective of health care provider stated Year value of money 1993 Sensitivity analysis performed for risk groups	–	*Prediction method*: incomplete of prediction method and method for comparator gives lower generalizability *Outcome to be predicted*: incomplete description of method for measurement, thresholds and criteria	No data on cost for prediction model, which will add to costs No discounting of costs	No presentation of costs related to effects As description of prediction method is limited it is questionable whether analysis is helpful for a decision‐maker
Zavras et al. (2000)	*Prediction method*: examination methods with radiography described, thresholds presented *Outcome to be predicted*: type of outcome and predictive performance for different risk‐groups presented	Perspective of private dental practitioner stated Discounting at different rates	Sensitivity analysis showed robust result	*Prediction method*: incomplete description, not possible to replicate *Outcome to be predicted*: incomplete description of method for measurement, thresholds and criteria Performance overestimated, based on high caries prevalence and on a study impossible to retrieve	Method not described to permit replication Costs based on fees with no reference Year value of money not presented	Results are not transferable to a setting with low caries prevalence As description of method for prediction is limited it is questionable whether analysis is helpful for a decision‐maker
Holst and Braune (1994)	*Prediction model*: definition and threshold presented *Outcome to be predicted*: type of outcome and predictive performance presented	–	Resource use estimated in minutes which may have higher generalizability than monetary cost	*Prediction model*: incomplete description of examination methods and vague description of care in comparative clinics *Outcome to be predicted*: incomplete description of method for measurement, thresholds and criteria. No radiography to confirm caries increment	No estimation of monetary cost Type of evaluation not stated Perspective not stated	No sensitivity analysis performed
Holst et al. (1997)	*Prediction model*: definition and threshold presented *Outcome to be predicted*: type of outcome and predictive performance presented bitewing radiography to confirm caries increment	–	Resource use estimated in minutes which may have higher generalizability than monetary cost	*Prediction model*: incomplete description of examination methods at baseline and follow‐up and vague description of care in comparative clinics *Outcome to be predicted*: incomplete description of method for measurement, thresholds and criteria	No estimation of monetary cost Type of evaluation not stated Perspective not stated	No sensitivity analysis performed
Higashi et al. (2002)	*Prediction method*: definition and threshold presented with data on prevalence of IL‐1 positive patients *Outcome to be predicted*: type of outcome and predictive performance presented	Markov model Cost for genetic test and laboratory fee presented Discounting at different rates Perspective of health care payer stated	Cost per QALY presented Sensitivity analysis performed Transferable to a general population around 35 years Helpful for a decision‐maker	*Prediction model*: prevalence of IL‐1 in Caucasians seem too low *Outcome to be predicted*: performance based on very high predictive estimates	Cost for interleukin genetics modeled with no reference Year value of money not presented	Not robust in regard to results of sensitivity analysis
Martin et al. (2014)	*Prediction method*: definition, examination methods and threshold presented *Outcome to be predicted*: criteria and thresholds described accurately Relevant long‐term follow‐up	Economic model described with reference Year value of money 2011		*Prediction model*: – *Outcome to be predicted*: method for measurement described in unpublished data and predictive performance based on referred data	Data on cost per tooth only for periodontal and prosthodontic treatment No data on cost for prediction model which will add to costs No perspective stated No discounting of data	No sensitivity analysis performed Results not transferable to a setting of men and women As description of method for prediction is limited it is questionable whether analysis is helpful for a decision‐maker

*Note*: Studies assessed using tool presented in Table 1 and results of appraisal summarized as strengths and weaknesses.

### 3.5 To what extent may the evaluations help decision‐makers to direct resources efficiently?

All studies showed that the cost‐effectiveness of the models or methods for the prediction of caries and periodontitis improved when the risk of caries or periodontitis is high. This finding is valuable for a decision‐maker; thus, the decision‐maker must consider the risk level in the target population before deciding on the effective methods. Apart from this finding, the studies are of quite low relevance for a decision‐maker, as they cannot easily be generalized or transferred to settings other than those studied.

## 4 DISCUSSION

This SR examined economic evaluation of multivariable models and methods for prognostic prediction of increased risk of caries and periodontitis. Following the search of the literature, six studies were considered eligible: four of increased risk of caries and two of increased risk of periodontal disease or tooth loss. The results of the SR highlighted a paucity of economic evaluations and revealed methodological shortcomings regarding the description of the predictive models/methods, and particularly of the economic evaluations.

### 4.1 Methodological considerations

In accord with the CDR's guidance (Centre for Reviews and Dissemination, 2009) the search strategy combined subject topic and economic terms. Other names for a prognostic prediction model or method in the dental literature include, for example, risk assessment, risk model and prognostic rules. These names were included in the searches and resulted in nonspecific searches. Most excluded publications merely presented assumptions of costs or cost‐effectiveness, indicating an interest in economic evaluation. Gray literature was not searched; instead, the reference lists of the included publications were searched, which is considered to be an effective way to identify relevant additional publications (Whiting et al., 2008). It is implied that MEDLINE, Embase and NHS EED cover the majority of relevant economic studies (Glanville & Paisley, 2010).

A major problem for the quality appraisal of economic evaluations lies in adapting methods to meet the challenges that arise as a result of the additional component of resources and costs to the prediction models and methods. As we did not find any published checklist for critical appraisal of prognostic prediction studies in combination with an economic evaluation, we designed and applied such a protocol and adapted it to the review questions.

### 4.2 Discussion of results

The result on paucity of studies was not unexpected, as the number of economic evaluations is also scarce regarding diagnostic methods (Christell et al., 2014). In other areas of oral health care, such as preventive and restorative interventions, significantly more health economic evaluations are found (Eow et al., 2019). One reason for the paucity of economic evaluations of prognostic predictive studies is probably that evidence is limited regarding the validity of existing prognostic predictive methods (Mejàre et al., 2014; Senneby et al., 2015) and multivariable prediction models (Cagetti et al., 2018; Tellez et al., 2013) to predict caries and caries development. Regarding periodontitis, a systematic review recently concluded that prediction models were deemed to be of “high risk” of bias due to the poor handling of data and the lack of validation (Du et al., 2018). Thus, valid and reliable data on the performance of models and methods applied for prognostic prediction are lacking. Evidence on the predictive performance of prognostic models and methods provides only part of the information needed for clinical decisions and subsequent interventions. Economic evaluations are increasingly used for evidence‐based decision making as an important component for planning health care delivery.

In line with the findings of a scoping review of economic evaluations in dental care (Eow et al., 2019), our SR revealed methodological limitations and heterogeneity among the studies. The disappointing levels of study design of the economic evaluation identified in our SR could be explained by the fact that most studies were published many years ago at a time when the methodology of economic evaluation in dental research was less developed. Generally, the critical appraisal of the included studies was hindered by a lack of clear reporting regarding the relation between costs and consequences. The generalizability of the findings into other settings with a different population and socioeconomic composition remains unclear. Whether the results of included studies are useful for a decision‐maker is questionable, mainly because the methods or models for the prediction and economic evaluation were incompletely described. The information of cost was limited, with none of the included studies presenting a detailed analysis in terms of identified, measured and valued resources used, as recommended by Husereau et al. (2013) Such a detailed approach in terms of a micro‐analysis would have furthered the understanding of the resource use and thus increased the usefulness of the analysis for a decision‐maker. Previous studies have shown that a systematic approach, structured in several successive steps can be helpful in analyzing all relevant costs (Christell, Birch, Horner, et al., 2012) and that the transferability of cost‐data between settings in different countries is limited, as the circumstances can differ greatly (Christell, Birch, Hedesiu, et al., 2012). The estimation of resource use in minutes, as presented by two included studies (Holst et al., 1997; Holst & Braune, 1994), may increase the transferability of the results to other settings. Finally, agencies for health technology assessment in different countries often require the results of cost‐effectiveness in terms of cost per QALY gained to enable comparisons between methods. Only one study, which assessed the prediction of periodontitis, presented the results in this way (Higashi et al., 2002).

Major challenges exist when conducting economic evaluations of prognostic prediction models and methods used clinically. There is a need for economic evaluations of prediction models and methods with various perspectives, well‐defined research questions, and measures of the cost and cost‐effectiveness.

## 5 CONCLUSIONS

Although prognostic methods and models for prediction of risk for caries or periodontitis are used daily in general dental practice, there is no evidence that it is cost‐effective to do so. This SR identifies a wide knowledge gap and highlights not only a paucity of economic evaluations of these models and methods but also reveals methodological shortcomings regarding the reporting in particular, of the economic evaluations. There is certainly a need for studies with well‐defined research questions, various perspectives and measures of cost‐effectiveness to identify key determinants of resource use to draw out how these determinants may be distributed within and between dental settings.

## AUTHOR CONTRIBUTIONS

Helena Fransson contributed to investigation, writing–review & editing, visualization, project administration. Thomas Davidson contributed to methodology, investigation, writing—review & editing. Madeleine Rohlin contributed to conceptualization, methodology, investigation, writing—original draft. Helena Christell contributed to methodology, investigation, writing–review & editing, visualization, project administration.

## CONFLICT OF INTEREST

All authors declare no conflict of interest.

## Supporting information


**Appendix** S1: Supporting Information

## Data Availability

The authors confirm that the data supporting the findings of this study are available within the article and its supplementary materials.
