# Supplementary material for: There is a paucity of economic evaluations of prediction methods of caries and periodontitis—A systematic review
Source: Clin Exp Dent Res. 2021 Feb 16;7(3):385–98. doi: 10.1002/cre2.405 (PMC8204028; doi:10.1002/cre2.405)
Supplement: Supplementary file 1 — Appendix S1: Supporting Information [file CRE2-7-385-s001.docx]

**Appendix 1. Supplementary Table 1**

Search strategy in MEDLINE 01.01.1965-04.09.2019, Web of Science 01.01.1986-19.09.2019, the Cochrane Library 01.01.1898-19.09.2019, the NHS Economic Evaluation Database (NHS EED) 01.01.1968-31.12.2014. Medline, Web of Science and Cochrane Library up-dated 02.09.2020.

| Database | Index terms |
| --- | --- |
| MEDLINE | "Periodontitis/analysis"[Mesh] OR "Periodontitis/anatomy and histology"[Mesh] OR "Periodontitis/classification"[Mesh] OR "Periodontitis/complications"[Mesh] OR "Periodontitis/diagnosis"[Mesh] OR "Periodontitis/diagnostic imaging"[Mesh] OR "Periodontitis/economics"[Mesh] OR "Periodontitis/enzymology"[Mesh] OR "Periodontitis/epidemiology"[Mesh] OR "Periodontitis/etiology"[Mesh] OR "Periodontitis/genetics"[Mesh] OR "Periodontitis/history"[Mesh] OR "Periodontitis/immunology"[Mesh] OR "Periodontitis/metabolism"[Mesh] OR "Periodontitis/microbiology"[Mesh] OR "Periodontitis/nursing"[Mesh] OR "Periodontitis/organization and administration"[Mesh] OR "Periodontitis/parasitology"[Mesh] OR "Periodontitis/pathology"[Mesh] OR "Periodontitis/physiology"[Mesh] OR "Periodontitis/physiopathology"[Mesh] OR "Periodontitis/psychology"[Mesh] OR "Periodontitis/statistics and numerical data"[Mesh] OR "Periodontitis/transmission"[Mesh] OR "Periodontitis/virology"[Mesh] OR "Dental Caries/analysis"[Mesh] OR "Dental Caries/anatomy and histology"[Mesh] OR "Dental Caries/chemistry"[Mesh] OR "Dental Caries/classification"[Mesh] OR "Dental Caries/complications"[Mesh] OR "Dental Caries/diagnosis"[Mesh] OR "Dental Caries/diagnostic imaging"[Mesh] OR "Dental Caries/economics"[Mesh] OR "Dental Caries/enzymology"[Mesh] OR "Dental Caries/epidemiology"[Mesh] OR "Dental Caries/etiology"[Mesh] OR "Dental Caries/genetics"[Mesh] OR "Dental Caries/history"[Mesh] OR "Dental Caries/immunology"[Mesh] OR "Dental Caries/metabolism"[Mesh] OR "Dental Caries/microbiology"[Mesh] OR "Dental Caries/nursing"[Mesh] OR "Dental Caries/organization and administration"[Mesh] OR "Dental Caries/parasitology"[Mesh] OR "Dental Caries/pathology"[Mesh] OR "Dental Caries/physiology"[Mesh] OR "Dental Caries/physiopathology"[Mesh] OR "Dental Caries/psychology"[Mesh] OR "Dental Caries/statistics and numerical data"[Mesh] OR "Dental Caries/transmission"[Mesh] OR "Dental Caries/virology"[Mesh]  AND  "Biofilms"[Mesh] OR "Biomarkers"[Mesh] OR dental caries activity tests[MeSH Terms] OR dental plaque[MeSH Terms]) OR food habits[MeSH Terms]) OR forecasting[MeSH Terms] OR hydrogen ion concentration[MeSH Terms] OR lactobacillus[MeSH Terms] OR microbiology[MeSH Terms] OR odds ratio[MeSH Terms] OR predict* OR predictive value of tests[MeSH Terms] OR prognosis[MeSH Terms] OR "Risk Assessment"[Mesh] OR risk factors[MeSH Terms] OR sensitivity and specificity[MeSH Terms] OR socioeconomic factors[MeSH Terms] OR streptococcus mutans[MeSH Terms] NOT cariostatic agents[MeSH Terms]  AND  "Delivery of Health Care"[Mesh] OR "Economics"[Mesh] OR "Health Resources/economics"[Mesh] OR "Health Services Research/economics"[Mesh] OR "Dental Health Services"[Mesh] OR "Models, Economic"[Mesh] OR "Outcome and Process Assessment (Health Care)"[Mesh] OR "Quality-Adjusted Life Years"[Mesh] OR "Quality of Life"[Mesh]) OR "Dentistry"[Mesh] OR "Decision Making"[Mesh] OR "Diagnosis"[Mesh]) OR "Oral Health"[Mesh]) OR "Radiology"[Mesh])OR "QALY") OR "Willingness to pay") OR "diagnosis") OR "Radiography") OR "radiology"  AND  "cost" OR "cost-benefit" OR "CEA" OR "cost-effective" OR "cost-effectiveness" OR "cost-utility" OR "cost-minimization" OR "health-economic evaluation" OR "health economics" AND  dent* OR dentistry OR "oral health"  OR  ("Risk"[Mesh] OR "Forecasting"[Mesh]) AND "Economics"[Mesh]) AND ("Periodontitis"[Mesh] OR "Dental Caries"[Mesh]) |
| Web of Science | ("Risk" OR "Forecasting") AND "Economics") AND ("Periodontitis" OR "Dental Caries") |
| The Cochrane Library | ("Risk"[Mesh] OR "Forecasting"[Mesh]) AND "Economics"[Mesh]) AND ("Periodontitis"[Mesh] OR "Dental Caries"[Mesh]) |
| NHS EED | (“Dentistry”) AND (Risk-assessment OR prediction) |

| **Appendix 2 Supplementary Table 2**  Excluded studies (n = 32) and reason for exclusion | | | |
| --- | --- | --- | --- |
| First author | Year | Country | Reason for exclusion |
| *Studies of caries* | | | |
| A.B Adams | 1995 | US | No data of predictive performance |
| J.D. Bader | 2008 | US | No analysis of cost |
| J. Cunha-Cruz | 2015 | US | Trial registration |
| X.L. Gao | 2010 | China | No analysis of cost |
| M. Hayes | 2017 | Ireland | No analysis of cost |
| M. Houpt | 2002 | US | No data of predictive performance |
| T.N. Imfeld | 1995 | Switzerland | No data of predictive performance |
| G. Koch | 1988 | Sweden | No analysis of cost |
| L. Li | 2014 | China | No data of predictive performance |
| N. Madan | 2011 | India | No data of predictive performance |
| M.E. Moss | 1995 | US | No data of predictive performance |
| F. Schwendicke | 2014 | Germany | No data of predictive performance |
| F. Schwendicke | 2015 | Germany | No data of predictive performance |
| F. Schwendicke | 2018 | Germany | No data of predictive performance |
| M. Tellez | 2015 | US | No analysis of cost |
| A. ter Pelkwijk | 1990 | The Netherlands | No analysis of cost |
| N. Tinanoff | 1995 | US | No analysis of cost |
| W.H. van Palenstein Helderman | 1989 | The Netherlands | No analysis of cost |
| E. Warren | 2010 | Australia | No data of predictive performance |
| E. Warren | 2016 | Australia | No data of predictive performance |
| *Studies of periodontitis* | | | |
| Z. Al Yahfoufi | 2015 | Lebanon | No data of predictive performance |
| M. Alsina | 2001 | Spain | No data of predictive performance |
| L.J. Brown | 2002 | USA | No data of predictive performance |
| P.E. Gjermo | 2009 | Norway | No data of predictive performance |
| D.F. Kinane | 2000 | UK | No data of predictive performance |
| J.S. Kinney | 2007 | USA | No analysis of cost |
| C.Y.Tsai | 2003 | USA | No data of predictive performance |
| S. Wignarajah | 2015 | Saudi Arabia | No data of predictive performance |
| *Studies of caries and periodontitis* | | | |
| M. Busby | 2013 | UK | No data of predictive performance |
| M. Busby | 2014 | UK | No data of predictive performance |
| C. Davenport | 2003 | UK | No data of predictive performance |
| I. Zickert | 2000 | Sweden | No data of predictive performance |
